# 305. Hospitalized Patients with Community-Acquired Pneumonia and Structural Lung Disease: A Multicenter Analysis

**DOI:** 10.1093/ofid/ofae631.095

**Published:** 2025-01-29

**Authors:** Geroge Doumat, Daniel Nielsen, David Ratz, Jennifer Horowitz, Elizabeth McLaughlin, Tawny Czilok, Anurag Malani, Danielle Osterholzer, Ashwin Gupta, Tejal N Gandhi, Lindsay A Petty, Louis Saravolatz, Scott A Flanders, Valerie Vaughn

**Affiliations:** Massachusetts General Hospital, Boston, Massachusetts; Michigan Medicine, Ann Arbor, Michigan; VA Ann Arbor Health System, Ann Arbor, Michigan; Michigan Medicine, Ann Arbor, Michigan; Michigan Medicine, Ann Arbor, Michigan; Michigan Medicine, Ann Arbor, Michigan; Trinity Health Michigan, Ann Arbor, Michigan; Hurley Medical Center/Michigan State University College of Human Medicine, Flint, Michigan; Michigan Medicine, Ann Arbor, Michigan; Michigan Medicine, Ann Arbor, Michigan; University of Michigan, Ann Arbor, Michigan; Michigan Medicine, Ann Arbor, Michigan; University of Michigan/Michigan Medicine, Ann Arbor, Michigan; University of Utah Medical School, Salt Lake City, UT

## Abstract

**Background:**

Patients with structural lung disease (SLD) are more susceptible to community-acquired pneumonia (CAP) and are at higher risk for resistant organisms. CAP studies often exclude patients with SLD making it difficult to determine when to treat patients empirically with broad-spectrum antibiotics (BSA). Here, we describe the epidemiology of CAP in inpatients with moderate to severe SLD.

**Figure:**
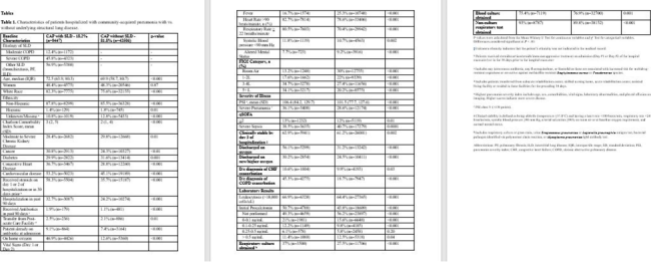

**Methods:**

Between 1/2017 and 12/2023, we analyzed data from inpatients with CAP in 68 Michigan hospitals. SLD was defined as documented moderate or severe chronic obstructive pulmonary disease, bronchiectasis, pulmonary fibrosis, or interstitial lung disease. BSA use was defined as therapy covering methicillin-resistant *Staphylococcus aureus* (MRSA) or *Pseudomonas* and was considered appropriate if recommended by IDSA/ATS CAP guidelines. General estimating equation models, adjusted for variables known to be associated with outcomes, were used to compare 30-day outcomes between SLD and non-SLD CAP inpatients. Logistic or Poisson models, as appropriate, were adjusted for variables known to be associated with the outcomes.

**Figure:**
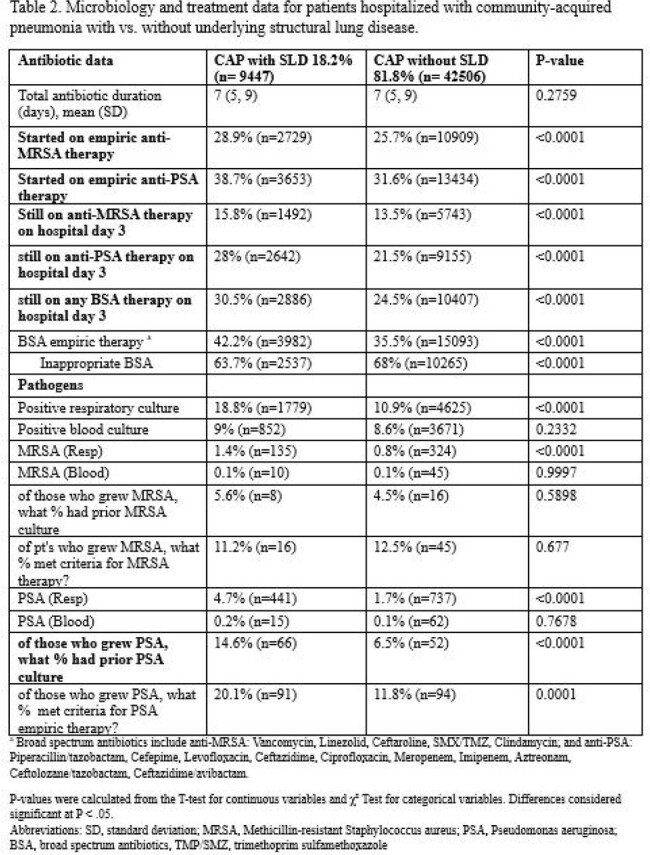

**Results:**

Of 51,953 patients with CAP across 68 hospitals, 18.2% (n=9,447) had documented SLD. Compared to patients without SLD, patients with SLD were sicker at baseline (see **Table 1** for details) and, despite lacking traditional risk factors, were more often found to have MRSA (1.4% vs. 0.8%, p< 0.01) or *Pseudomonas aeruginosa* (4.7% vs. 1.7% p< 0.01) in their respiratory cultures (**Table 2**). While 42.2% of patients with SLD (vs. 35.5% without SLD) received broad-spectrum antibiotics, only 16.1% (vs. 10% without SLD) of such therapy was recommended by guidelines. Compared to patients without SLD, those with SLD had higher odds of mortality (aOR=1.31, p< 0.01 ) and hospital readmission (aOR = 1.19, p< 0.01; **Table 3**).

**Figure:**
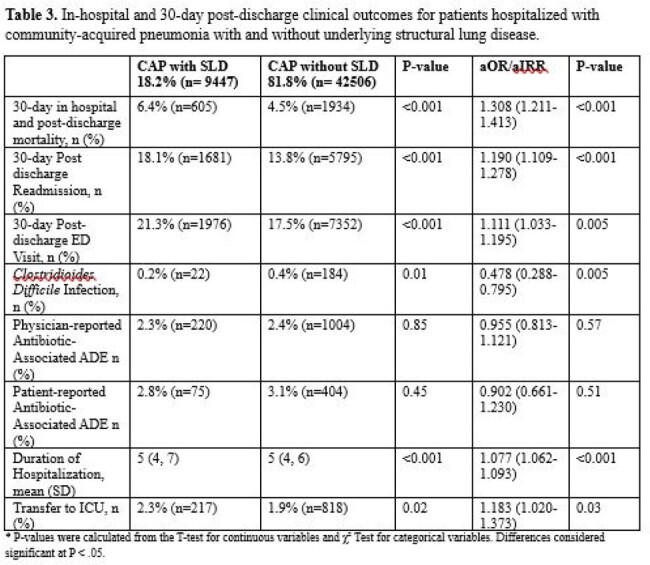

**Conclusion:**

Patients with CAP and underlying SLD had more severe disease and experienced worse clinical outcomes. Though they were more likely to grow MRSA or *Pseudomonas* than patients without SLD, patients with SLD were far more likely to have non-guideline concordant empiric BSA use. New models may be needed to identify the risk of resistant pathogens in SLD and support better selection of empiric therapy.

**Disclosures:**

**Elizabeth McLaughlin, MS, RN**, Blue Cross Blue Shield of Michigan: Salary Support

